# Transcriptome Analyses of the Anti-Proliferative Effects of 20(S)-Ginsenoside Rh2 on HepG2 Cells

**DOI:** 10.3389/fphar.2019.01331

**Published:** 2019-11-07

**Authors:** Ji Zhang, Weibo Li, Qiaoyun Yuan, Jing Zhou, Jianmei Zhang, Yufeng Cao, Guangbo Fu, Weicheng Hu

**Affiliations:** ^1^Jiangsu Collaborative Innovation Center of Regional Modern Agriculture & Environmental Protection/Jiangsu Key Laboratory for Eco-Agricultural Biotechnology around Hongze Lake, School of Life Sciences, Huaiyin Normal University, Huaian, China; ^2^Department of Urology, The Affiliated Huaian No. 1 People’s Hospital of Nanjing Medical University, Huaian, China

**Keywords:** 20(S)-ginsenoside Rh2, hepatocellular carcinoma, anti-proliferation, p53 signaling pathway, RNA-seq

## Abstract

20(*S*)-ginsenoside Rh2 (Rh2), a well-known protopanaxadiol-type ginsenoside from *Panax ginseng* has especially gained attention for its anticancer activities on various types of human cancer cells. However, the molecular mechanism through which Rh2 promotes apoptosis in hepatocellular carcinoma (HePG2) cells is not known at the transcriptome level. Rh2 can specifically inhibit the proliferation of HePG2 in a dose- and time-dependent manner. Moreover, Rh2 can significantly increase the apoptosis which was related with an increase in protein expression levels of caspase-3, caspase-6, and poly (ADP-ribose) polymerase. Comparison of RNA-seq transcriptome profiles from control group and Rh2-treated group yielded a list of 2116 genes whose expression was significantly affected, which includes 971 up-regulated genes and 1145 down-regulated genes. The differentially expressed genes in p53 signaling pathway and DNA replication may have closely relationships to the cells apoptosis caused by Rh2 treatment. The results of qPCR validation showed that dynamic changes in mRNA, such as CDKN1A, CCND2, PMAIP1, GTSE1, and TP73.

## Introduction

Ginseng (*Panax ginseng* C.A.Mey) has been known as the "King of Herbs" since ancient times in eastern Asia (Hossen et al., 2017). It is a perennial herb that grows in cool habitats and belongs to the Araliaceae family (Park and Cho, 2009). The root of ginseng been widely used in traditional medicine in eastern Asian countries to promote health (Yu et al., 2015). Ginsenosides are the main essential bioactive ingredients that have various pharmacological activities, including anti-inflammatory, anti-allergic, anti-fatigue, anti-stress, and anti-cancer activities (Oh et al., 2014). More than 100 ginsenosides from *Panax* species have been identified and categorized into four types: protopanaxadiol, protopanaxatriol, oleanolic acid, and ocotillol-type (Nag et al., 2012). Ginsenoside Rh2 is a metabolite produced by Rg3, Rb1, Rb2, and Rc by human intestinal bacteria after absorption (Chi et al., 2005). Ginsenoside Rh2 is classified into 20(*S*) and 20(*R*) forms according to the different arrangement of the hydroxyl group at the C-20 position, and 20(*S*)-Rh2 was found to possess strong cytoxic activities to several cancer cells (Liu et al., 2010; Kim et al., 2017). 20(*S*)-Rh2 has a number of pharmacological properties, such as anti-inflammatory, anticancer, cardioprotection, and neuroprotective effects (Zhang et al., 2012). Rh2 has especially gained attention for its broad-spectrum anti-proliferative effect on a variety of human cancer cells (Oh et al., 1999; Kim et al., 2004; Qi et al., 2011; Guo et al., 2014; Shi et al., 2014; Qian et al., 2016; Yang et al., 2016; Wang et al., 2018). In addition, additional *in vivo* work showed that Rh2 also can significantly suppress the growth of uterine leiomyoma in SD rats (Zhu et al., 2015). The tumor growth inhibitory activity of Rh2 was also found in H22 tumor-bearing mice (Chen et al., 2017) and nude mice bearing human ovarian cancer cells (Nakata et al., 1998).

The molecular pathways affected by Rh2 in cancer cells are mainly through apoptosis-related pathways. for example, Rh2 induces apoptosis in colorectal cancer cells through activation of p53 (Li et al., 2011). in human leukemia Reh cells, Rh2 could induce apoptosis through the mitochondrial pathway (Xia et al., 2014). Rh2 also suppresses proliferation of hepatocellular carcinoma (HCC) cells by targeting EZH2 to regulate CDKN2A-2B gene cluster transcription (Li et al., 2017). Although apoptosis and other related pathways and genes have been identified in Rh2-treated cancer cells, the molecular mechanism through which Rh2 promotes cancer cell apoptosis is not known at the transcriptome level. To the best of our knowledge, this study is the first transcriptome analysis of the anti-proliferative effects of Rh2 on hepg2 cells. The results will aid the identification of functional genes and provide a more comprehensive understanding of the role of Rh2 in promoting apoptosis in human hematoma cells.

## Materials and Methods

### Materials

20(*S*)-Rh2 was obtained from Chengdu Must Bio-Technology Co., Ltd. (Chengdu, Sichuan, China) and the purity (> 98%) was determined by high-performance liquid chromatography. 3-4,5-Dimethylthiazol-2-yl)-2,5-diphenyltetrazolium bromide (MTT) was obtained from Sigma-Aldrich (St. Louis, MO, USA). The fetal bovine serum was obtained from Corning (Medford, MA, USA). Penicillin–streptomycin solution (10,000 unit/10,000 µg/ml) was purchased from Invitrogen–Gibco (Carlsbad, CA, USA). Minimum essential medium was obtained from Sigma-Aldrich (Poole, UK). Protease and phosphatase inhibitors cocktail tablets were bought from Roche (Mannheim, Germany). Phosphate buffered saline (PBS) tables was purchased from Amresco (Solon, OH, USA). The antibodies for caspase-3, -6, and poly (ADP-ribose) polymerase (PARP) were obtained from Cell Signaling Technology (Beverly, MA, USA). Trizol reagent was purchased from Ambion (Austin, TX, USA). Reverse transcription kit was bought from Invitrogen (Carlsbad, CA, USA). Luna universal qPCR master mix was obtained from New England BioLabs (Ipswich, MA, USA). Annexin-PI kit was obtained from BD Biosciences (San Jose, CA, USA). All cell culture suppliers were purchased from Coster (Cambridge, MA, USA).

### Cell Line and Cell Culture

The HepG2 cell line was purchased from the Cell Bank of Type Culture Collection of the Chinese Academy of Sciences (Wuhan, China) and cultured in minimum essential medium supplemented with 10% fetal bovine serum, 100 U/ml penicillin, and 100 µg/ml streptomycin at 37°C in a humidified atmosphere of 95% air–5% CO_2_ in an humidified CO_2_ incubator (Heracell 150i, Thermo Fisher Scientific, Waltham, MA, USA).

### Cell Viability

The cytotoxicity of Rh2 on HepG2 cells was determined using the MTT assay as described previously (Bai et al., 2019). Briefly, cells were seeded (1 × 10^4^ cells/well) in 96-well plate for overnight. Then the cells were incubated with an increasing concentration of Rh2 (0–60 µM) for 24 and 48 h, respectively. The supernatant was removed *via* aspiration and 100 µl of MTT solution (0.5 mg/ml) was added and cells were continuously cultured until 100 µl of MTT stop solution (10% SDS, 0.01 M hydrochloric acid) was added to each well to solubilize the formazan. The absorbance was measured at 550 nm using a microplate reader (Tecan Infinite M200 Pro, Männedorf, Switzerland).

### Cell Morphology Analysis

To examine the effect of Rh2 on the morphology of HepG2 cells, cells (5 × 10^4^ cells/well) were seeded on sterilized cover glasses in 6-well plate for overnight. Then the cells were incubated with Rh2 (20 or 50 µM) for 24 h and the cell morphology was observed with Olympic (Tokyo, Japan). The changes of microstructure were surveyed under scanning electron microscope (SEM). Treated cells were washed with PBS three times and fixed with 2.5% glutaraldehyde solution for 1 h at room temperature. Subsequently, cells were rinsed again with PBS three times, and then dehydrated using graded ethanol (30%, 50%, 70%, 90%, 100%). Cells were fixed onto a copper stub and coated with gold using a sputter coater under vacuum, and then the surface morphology of cells was characterized by SEM (FEI Quanta 450 FEG, Hillsboro, USA).

### Annexin V-Fitc Apoptosis Assay

The cells for apoptosis were examined using Annexin V-FITC apoptosis detection kit. Cells (4 × 10^5^ cells/well) were seeded in 6-well plate for overnight. Then the cells were incubated with Rh2 (20 or 50 µM) for 24 h. After treatment, the cells were collected by trypsinization with 0.25% EDTA and centrifuged at 1500 rpm for 3 min to remove the medium. Then the precipitation was washed with pre-cold PBS and resuspended in 100 µl 1 × binding buffer containing 5 µl FITC-Annexin V and 5 µl propidium iodide. After incubation in the dark for 15 min, the fluorescence intensity was evaluated under an Accuri C6 Plus flow cytometer (Becton, Dickinson and company, CA, USA).

### RNA Extraction, Library Construction, and Sequencing

HepG2 cells were seeded in 40 mm dishes at a density of 1 × 10^6^ cells/well. Following treatment with 50 µM Rh2 for 24 h, the cells were washed twice with cold PBS and collected by centrifuged for 3 min. Total RNA of each sample was extracted with a Trizol reagent kit (Invitrogen). RNA quality was assessed on an Agilent 2100 Bioanalyzer (Agilent Technologies, Palo Alto, CA, USA) and checked by agarose gel electrophoresis. After enrichment of mRNA using Oligo(dT) beads, it was fragmented into short fragments in fragmentation buffer and reverse transcripted into cDNA with random primers. Then, DNA polymerase I, RNase H, dNTP, and buffer were used to synthesize the second-strand cDNA. The synthesized product were purified with a PCR purification kit (Qiagen, Venlo, The Netherlands), end repaired, poly(A) added, and ligated to Illumina sequencing adapters. After that, the ligated products were size selected by agarose gel electrophoresis, PCR amplified, and sequenced with Illumina HiSeq2500 (Gene Denovo Biotechnology Co. Guangzhou, China).

### RNA-Seq Data Analysis

In order to ensure the quality of the data for the following analysis, the raw data of six samples were firstly processed through in-house perl scripts. In this step, clean data were obtained by removing reads containing adapter, reads containing ploy-N, and low quality reads from raw data. At the same time, Q20, Q30, and GC content of the clean data were calculated. All the downstream analyses were based on the clean data with high quality.

### Differentially Expressed Genes

RNAs differential expression between control and Rh2 treated groups was analyzed using DESeq2 software (Love et al., 2014). We defined the genes with false discovery rate (FDR) < 0.05 and absolute fold change (FC) ≥ 2 as differentially expressed genes (DEGs).

### Gene Ontology and Pathway Enrichment Analysis

Gene Ontology (GO) is a widely used ontology in the bioinformatics field, which offers a semantic vocabulary standard to define and describe the functions of genes and proteins, and can be updated and applied in any organism (Myhre et al., 2006). Hierarchical system was used in GO for gene classification and putting identical genes at the same level. The vocabulary of genes and proteins involved in GO covers three aspects of biology: molecular function (MF), cellular component (CC), and biological process (BP). Terms is the basic units of GO, which were also known as GO classes. Each GO term has a human readable name and a GO ID, and belongs to one of the three sub-ontologies.

Genes often interact with other genes when performing their functions in BP. We often classify genes involved in same biological functions into pathways, which are helpful for us to further understand the biological functions of genes. Kyoto Encyclopedia of Genes and Genomes (KEGG) is a pathway-related database resource for understanding high-level functions and utilities of the biological systems (Lin and Feng, 2012). KEGG pathway enrichment analysis in DEGs comparing with the whole genome background can identify DEGs into significantly enriched metabolic pathways or signaling pathways.

In the present study, we use the online DAVID functional annotation clustering tool (https://david.ncifcrf.gov) by choosing the default option to determine the significantly represented GO categories and KEGG enriched pathways for DEGs of HepG2 cells response to Rh2 treatment. The calculated *p* value was corrected with FDR ≤0.05 as a threshold under the FDR correction. At the same time, GO terms and KEGG Pathways of DEGs meeting this condition were defined as significantly enriched GO terms and KEGG pathways.

### Real Time Quantitative RT-PCR

HepG2 cells were seeded in 40 mm dishes at a density of 1 × 10^6^ cells/well. Following treatment with 50 µM Rh2 for 24 h, the RNA extraction was carried out as described in the section of RNA extraction, library construction, and sequencing. A 20 µl PCR reaction system was consist with 2 µl cDNA, 10 µl SYBR mixture, 1 µl forward primer, 1 µl reverse primer, and 6 µl deionized water. After mixing, the PCR reaction was performed using CFX-96™ Real-Time instrument (Bio-Rad, CA, USA). The GAPDH gene was used as a house keeping gene to normalize the expression level of the test genes, and the relative gene expression level was analyzed using the 2^−ΔΔCT^ method. All of the samples were analyzed in triplicate. Primers were synthesized by Sangon Biotech (Shanghai, China) and were listed in **Table 1**.

**Table 1:** Primers used for RT-qPCR in this study.

Gene name	Forward primer (5′–3′)	Reverse primer (5′–3′)
PMAIP1	AGGAGGTGCACGTTTCATCA	ACAGTAGGCCAGCGGTAATC
SESN1	GGCAAGACATCAGTGCTCCT	AGCCGAATGTGAATGAGGCA
TP73	CACACCATCACCATCCCCAA	ACACAGGAAGGAGAGGGGAG
GTSE1	GCAAGTCAGGCAGAATGGGA	CAGGAATCTCGCCGTCTGAT
CCNE1	GGGTATCAGTGGTGCGACAT	TGCTCTGCTTCTTACCGCTC
TP53I3	CTGAACCGGGCGGACTTAAT	CTGGGATAGGCATGAGGAGC
CDKN1A	TCCTCATCCCGTGTTCTCCT	CACCCTGCCCAACCTTAGAG
CCND3	ACTGTGCATCTACACCGACC	TGTAGCACAGAGGGCCAAAA
CCND2	AAAGGAAGGAGGTCAGGGGA	TCCTTCTGCACGCACTTGAA
BBC3	CATACTGGACTCCCAGCCCT	GAAGGAGCACCGAGAGGAGA
DDB2	AAACGCCCAGAAACCCAGAA	GACAGATGGCCAGGAAGCTC
GAPDH	TGGAAGGACTCATGACCACA	TTCAGCTCAGGGATGACCTT

### Western Blot Analysis

HepG2 cells were seeded in 40 mm dishes at a density of 1 × 10^6^ cells/well. Following treatment with 20 or 50 µM Rh2 for 24 h, the cells were washed twice with cold PBS and centrifuged for 3 min, and the supernatant was removed. The cells were lysed in 200 µl of lysis buffer and protein content was measured with Bradford reagent using bovine serum albumin as a standard. Equal amounts of protein were resolved on 10%–15% SDS-polyacrylamide gels for 2 h at 120 V and then transferred to PVDF membranes. The membranes were blocked for 2 h using 5% milk powder in Tris-buffered saline containing Tween 20 (1 × TTBS). After incubation, the membranes were washed with 1 × TTBS and incubated overnight with the primary antibody (1:1000). The membranes were washed and incubated for 1 h with horseradish peroxidase-conjugated secondary anti-IgG (1:500). The protein bands were visualized using the eECL Western Blot Kit (CWBIO, China) according to the manufacturer's instructions and photographed using the Tanon 5200 Multi imaging system (Tanon,Shanghai, China).

### Statistical Analysis

The results have been represented as the means standard deviation (SD). The significance was analyzed using SPSS 20.0 package (SPSS Inc., Chicago, IL, USA). Differences among samples were compared using a Duncan's multiple range test and *p* values less than 5% were considered to be statistically different.

## Results and Discussion

### Rh2 Inhibits HepG2 Cell Proliferation

HCC is the most malignant primary human liver cancer and has a poor outcome after combined surgical treatment, radiotherapy, and chemotherapy (Lin, 2014). Although our understanding of the mechanism of HCC carcinogenesis has improved, the prognosis of HCC remains poor. Hence, there is an urgent need to develop effective therapies for HCC (Shang et al., 2016). There is growing interest in developing more effective therapeutic agents from natural resources.

**Figure 1:**
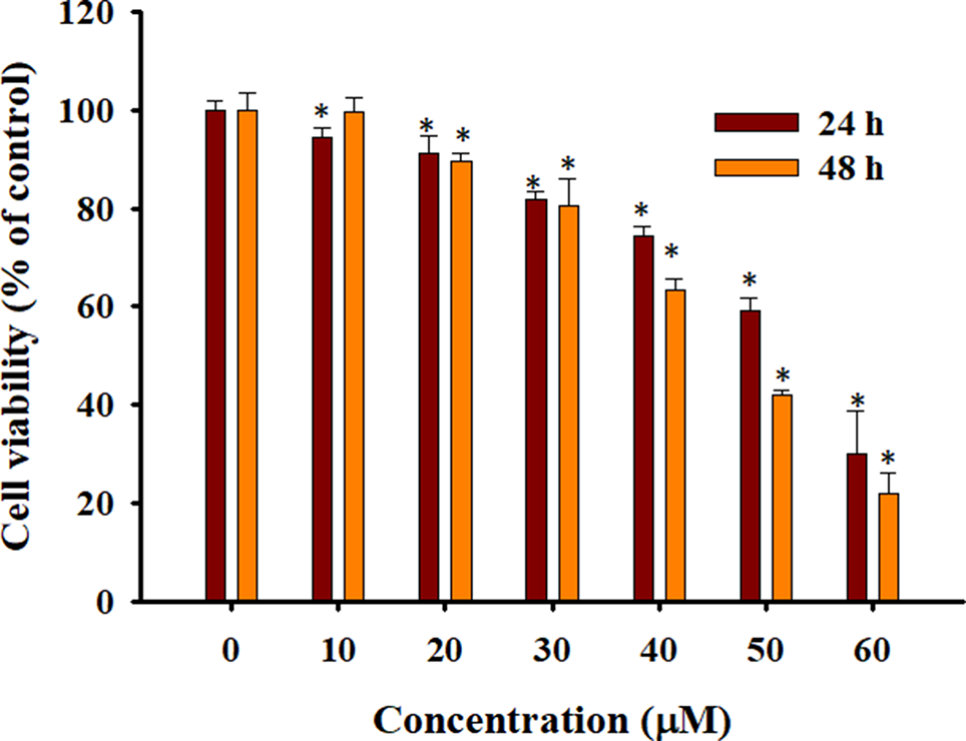
Cytotoxicity of 20(S)-ginsenoside Rh2 (Rh2) on HepG2 cells. Cells were treated with various concentrations of Rh2 for 24 or 48 h and cell viability was evaluated using the 3-4,5-dimethylthiazol-2-yl)-2,5-diphenyltetrazolium bromide (MTT) assay. The values are presented as the mean ± SD (n = 3). Within one column,*p < 0.05 vs. control group.

**Figure 2:**
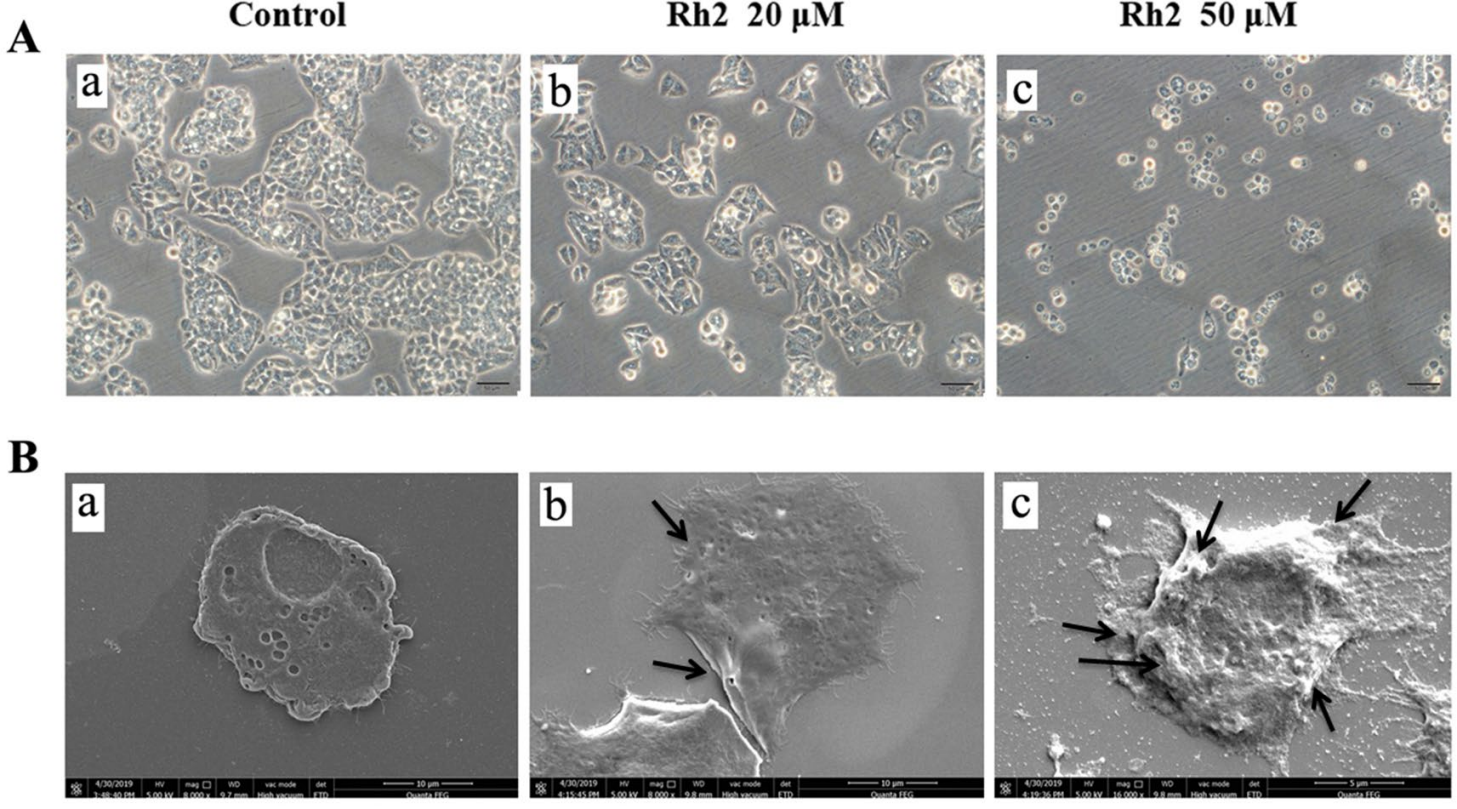
Photomicrographs of HepG2 cells after incubation with Rh2 under a light microscope **(A)** and SEM **(B)**: (a) control, (b) 20 µM Rh2 treatment, and (c) 50 µM Rh2 treatment. Arrows indicated the damage cell membrane.

To confirm the effects of Rh2 on HepG2 human hepatocarcinoma cells, we performed an MTT assay (**Figure 1**) and found that Rh2 inhibited HepG2 cell growth in a concentration-dependent manner with an IC_50_ of 51.97 and 45.46 µM at 24 and 48 h, respectively, which is consistent with the literature (Chen et al., 2015; Shi et al., 2016). To confirm the cytotoxic effects of Rh2 on HepG2, cell morphology was visualized under an inverted microscope and by SEM. As shown in **Figure 2A**, the untreated cells were spread out and flattened, whereas the Rh2-treated cells were round, shrunken, and showed altered adherence. Moreover, cell numbers were reduced. As shown in **Figure 2B**, control cells had a normal cell architecture with a distinct cytoskeleton, whereas the Rh2-treated cells had atypical morphological changes, including cell shrinkage and incomplete membranes.

### Rh2 Induces Apoptosis in HepG2 Cells

Apoptosis, also known as programmed cell death, is an important regulatory mechanism by which the body maintains cell structure and normal tissue development (Bennetts and Pierce, 2010). Inducing tumor cell apoptosis is an important way to inhibit tumor growth. Rh2 inhibits HCC cells in many ways, including inhibiting cell proliferation, inducing cell differentiation, promoting cell apoptosis, inhibiting cell invasion and metastasis, reducing drug resistance, and improving immunity (Li et al., 2017). Although the anti-HCC cell activity of Rh2 has been extensively reviewed, only a few studies have investigated the molecular responses in HCC HepG2 cells (Li et al., 2011; Cheong et al., 2015; Chen et al., 2016; Shi et al., 2016). In this study, we investigated the anti-cancer effects of Rh2 on HepG2 cells and the molecular mechanism by which Rh2 promotes HepG2 cell apoptosis at the transcriptome level.

**Figure 3:**
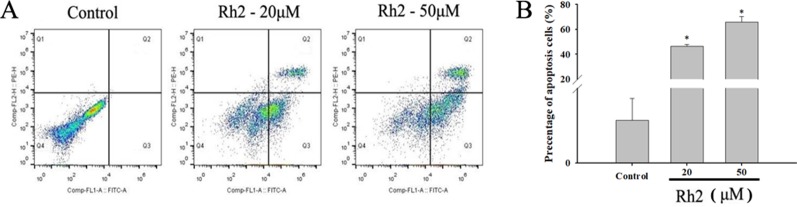
Effects of Rh2 on cell apoptosis in HepG2 cells. **(A)** Cells were treated with various concentrations of Rh2 for 24 h and the apoptosis rate was determined using Annexin V-FITC/PI staining. **(B)** Histogram showing the percentage of apoptotic cells. The values are presented as the mean ± SD (n = 3). *p < 0.05 vs. control group.

**Figure 4:**
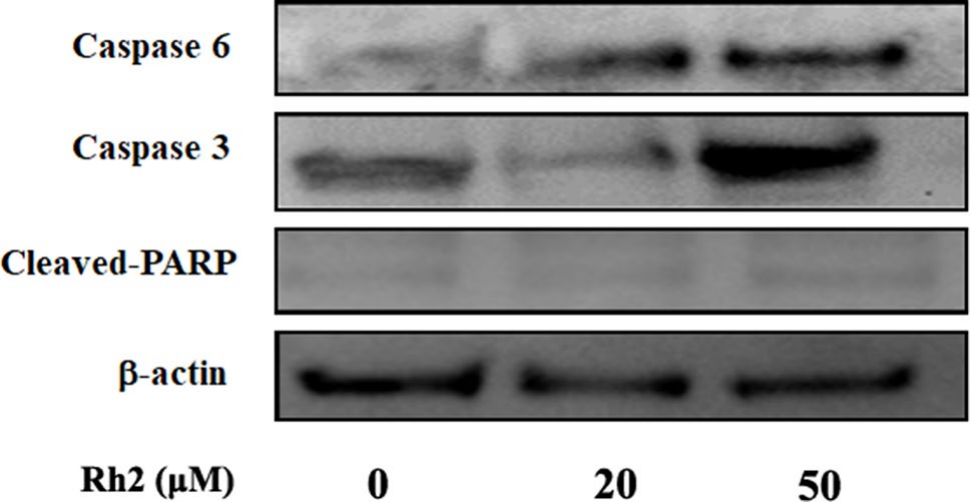
The effects of Rh2 on apoptosis-related proteins expression in HepG2 cells. Cells were treated with various concentrations of Rh2 for 24 h. After preparing the total protein, caspases 3 and 6, and cleaved-PARP were measured *via* Western blotting.

To confirm the increased apoptotic effects of Rh2 on HepG2 cells, the cell lines were treated with 20 and 50 µM Rh2 for 24 h. After treatment, the cells were examined for apoptosis *via* flow cytometry using Annexin V-FITC/PI double staining (**Figure 3A**). The percentage apoptosis increased from 0.057 in the control group to 46.5% and 66.07% (**Figure 3B**) at 20 and 50 µM, respectively. These results indicate that the anti-proliferative effects of Rh2 on HepG2 cells are mostly cytostatic, rather than cytotoxic. Caspases are a family of cysteine proteases and the primary drivers of apoptotic cell death, and play a critical role in the transduction of apoptosis signals (Vaculova and Zhivotovsky, 2008). Among the major caspases, caspases 3 and 6 are executioners of tumor growth and progression. PARP also plays important roles in apoptosis, the DNA damage response, and cell cycle regulation (Smulson et al., 2000). Interestingly, apoptosis is a genetically regulated cell suicide program mediated by activation of effector caspases 3 and 6. The effects of Rh2 on the expressions of apoptosis-related proteins were determined by assessing the expression levels of caspases 3 and 6 and PARP protein in HepG2 cells *via* Western blotting. As shown in **Figure 4**, treatment with 50 µM Rh2 dramatically increased caspase 3 and 6 expression in HepG2 cells but had less of an effect on cleaved-PARP protein. These results indicate that Rh2 promotes the apoptosis of hepatoma cells by upregulating the expression of caspase family proteins.

### Data Generation and Comparison With RNA-Seq Analyses

To identify what genes were regulated *via* Rh2 treatment, and further elucidate the molecular mechanism by which Rh2 inhibits HepG2 cell proliferation, transcriptome analyses were carried out on Rh2-treated and control HepG2 cell samples. Illumina HiSeq2500 was used for sequencing. As shown in **Table 2**, there were 70,625,504 (99.77%), 60,206,338 (99.78%), and 66,661,250 (99.79%) clean reads in control groups CK-1 to -3 and 60,916,030 (99.81%), 62,125,098 (99.78%), and 67,314,974 (99.76%) in Rh2-treated groups 1–3, respectively. The Q30 proportion of all six samples exceeded 93%, indicating that the quality of the clean reads obtained was high, and the results met the requirement for the next step in bioinformatics analyses. The rates of clean reads for all six samples exceeded 97%, indicating that the utilization rate of sequencing data was high.

**Table 2:** Data quality and reference sequence alignment analysis results.

Summary	CK-1	CK-2	CK-3	Rh2-1	Rh2-2	Rh2-3
N	3500 (0.00%)	2966 (0.00%)	3296 (0.00%)	3038 (0.00%)	2810 (0.00%)	3030 (0.00%)
Adapters	29436 (0.04%)	26482 (0.04%)	23684 (0.04%)	20490 (0.03%)	26500(0.04%)	31116 (0.05%)
Low quality	126,352 (0.18%)	105,916 (0.18%)	111,256 (0.17%)	92,540 (0.15%)	107,442 (0.17%)	126,640 (0.19%)
Clean	70,625,504 (99.77%)	60,206,338 (99.78%)	66,661,250 (99.79%)	60,916,030 (99.81%)	62,125,098 (99.78%)	67,314,974 (99.76%)
Clean base	10.57G	9.01G	9.97G	9.11G	9.30G	10.07G
GC (%)	51.53%	51.88%	52.22%	52.53%	52.39%	51.77%
Q30 (%)	93.98%	94.12%	94.13%	94.39%	93.92%	94.08%
Genome map rate of clean reads	97.11%	97.18%	97.21%	97.18%	97.07%	96.95%
Unique match in genome mapped reads	93.96%	94.04%	94.06%	93.73%	93.62%	93.47%
Multiple match in genome mapped reads	3.16%	3.14%	3.16%	3.45%	3.45%	3.48%

### DEG Response to Rh2 Treatment

**Figure 5:**
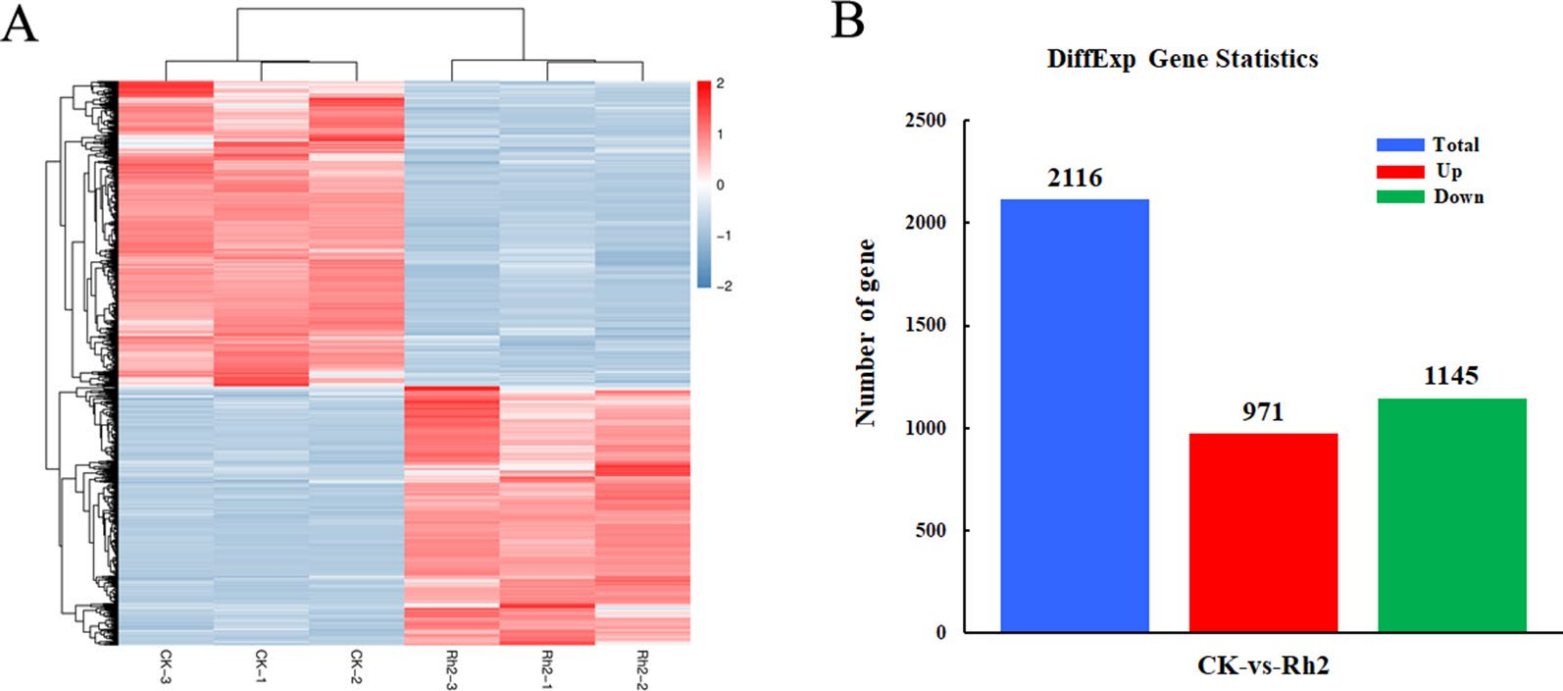
Differentially expressed genes between control and Rh2-treated cells using a heat map **(A)** and the distribution pattern **(B)**.

To identify the DEG response to Rh2 treatment, the differential expression multiple between different samples was determined from the level of gene expression. Using FC ≥ 2 and FDR < 0.05 as criteria, 2116 DEGs were identified between the control and Rh2-treated groups. Next, the DEG clusters were analyzed in a heat map (**Figure 5A**). DEGs identified in biological replicates clustered together, indicating good reproducibility of the treatments. The DEG statistics indicated 971 upregulated genes and 1145 downregulated genes (**Figure 5B**).

### GO Enrichment Analyses of DEGs

**Figure 6:**
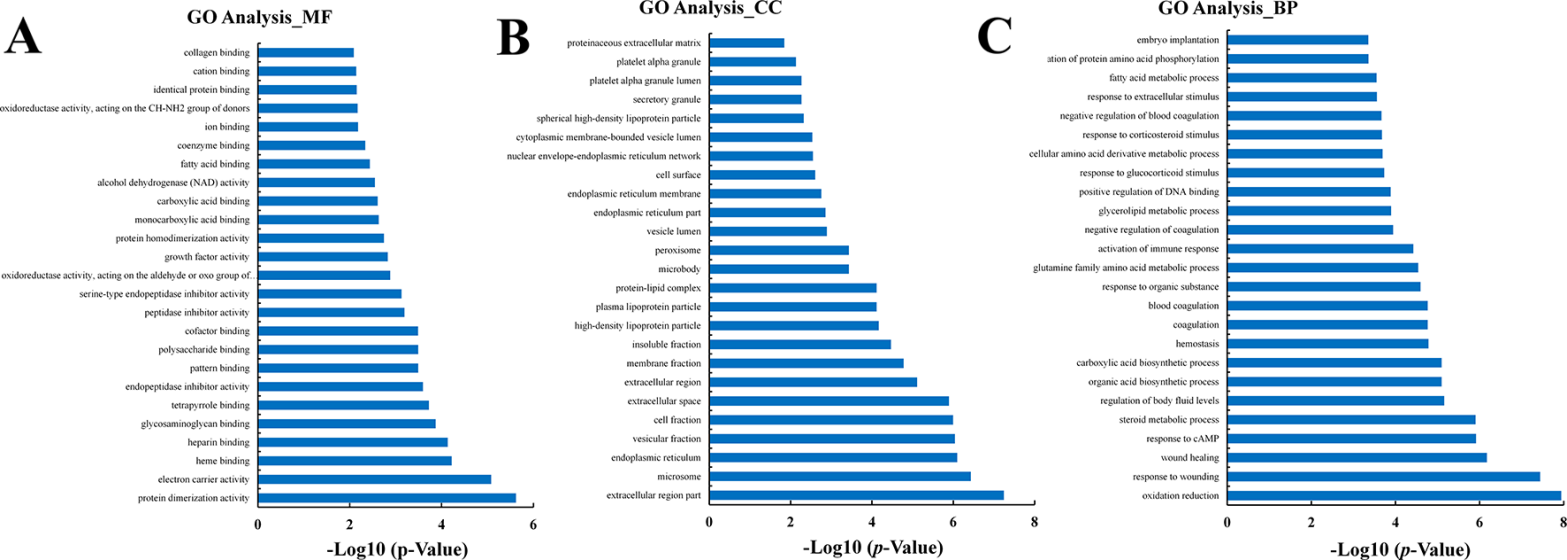
The classification of Gene Ontology (GO) functional enrichment analyses with the differentially expressed genes (DEGs) for CK vs. Rh2. The top 25 functional enriched classes of DEGs annotated by molecular function (MF) **(A)**, cell components **(B)**, and biological processes (BP) **(C)** sub-ontology of GO enrichment analyses.

The DEGs in the CK vs. Rh2 groups were analyzed for GO functional enrichment. Each group of DEGs was annotated into three classifications: MF, CC, and BP. The top 25 functional enriched classes of each group are shown in **Figure 6**. In MF, the DEGs were mainly enriched for protein dimerization activity, electron carrier activity, heme binding, and heparin binding (**Figure 6A**). In CC, the DEGs were mainly enriched in the extracellular region part, microsome, endoplasmic reticulum, and vesicular fraction (**Figure 6B**). In BP, the DEGs were mainly enriched in oxidation reduction, response to wounding, wound healing, and response to cAMP (**Figure 6C**).

### KEGG Pathway Enrichment Analyses of DEGs

**Figure 7:**
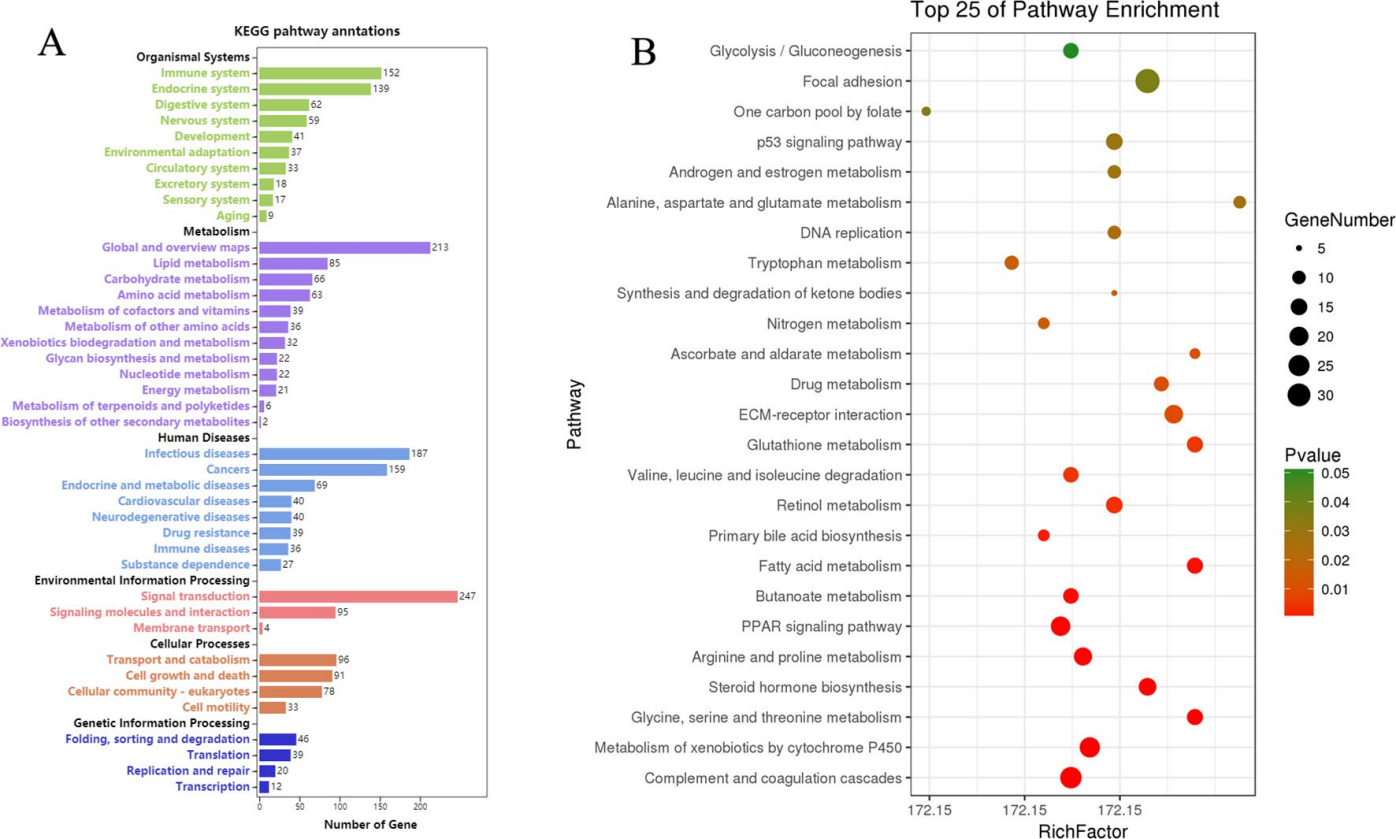
Kyoto Encyclopedia of Genes and Genomes (KEGG) pathways enrichment analyses of DEGs for CK vs. Rh2. **(A)** KEGG pathway annotation and classification. **(B)** Scatterplot of the top 25 pathways in KEGG enrichment.

KEGG pathway analyses of the DEGs in CK vs. Rh2 were performed online at OmicShare. Pathways and all of the DEGs clustered into six categories: organismal systems, metabolism, human diseases, environmental information processing, cellular process, and genetic information processing (**Figure 7A**). The environmental information processing category includes signal transduction, signaling molecules and interaction, and membrane transport. The cellular processing category includes transport and catabolism, cell growth and death, cellular community-eukaryotes, and cell motility. A scatterplot was used to show the top signaling pathways in the KEGG enrichment analyses. As shown in **Figure 7B**, the apoptosis-related signaling pathways, including the p53 signaling pathway and DNA replication, were significantly enriched. The DEGs in these two pathway categories may have close relationships to the cell apoptosis caused by Rh2 treatment, highlighting the modulation of Rh2 in tumorigenesis

### DEGs in Apoptosis and Cell Cycle-Related Pathways

Transcription factor p53 is regulated by its phosphorylation (Zhang et al., 2002). p53 is the upstream regulator of caspase family proteins in oxidative stress and DNA damage induced by various stress conditions (Vakifahmetoglu et al., 2006). The function of p53 is to induce mitochondria to release cytochrome C, which directly improves caspase 9 activity (Schuler et al., 2000). The activation of caspase 3 is promoted by the activation of caspase 9 (Kaushal et al., 2004). The p53-mediated apoptotic signaling pathway is important for cancer treatment with multiple chemotherapies (Zhan et al., 2014). Recent studies have shown that various natural products can induce apoptosis, which is closely associated with the p53 pathway (Laux et al., 2004; Yeruva et al., 2005; Volate et al., 2010). **Table 3** lists the DEGs in KEGG pathways related to cell apoptosis and the cell cycle. Fifteen DEGs were annotated in the p53 signaling pathway: six were upregulated and nine were downregulated.

**Table 3:** DEGs in apoptosis-related pathways.

Pathway term	Symbols of genes belong to each pathways	
	Upregulated genes	Downregulated genes
DNA replication	/	LIG1, PCNA, POLA2, MCM2, MCM3, RNASEH2A, MCM4, MCM5, MCM6, RPA3
p53 signaling pathway	PMAIP1, CDKN1A, CCND2, BBC3, SERPINE1, IGFBP3	SESN1, TP73, GTSE1, CCNE1, TP53I3, CCND3, DDB2, SESN3, RRM2

### Differential Expression of Representative DEGs

**Figure 8:**
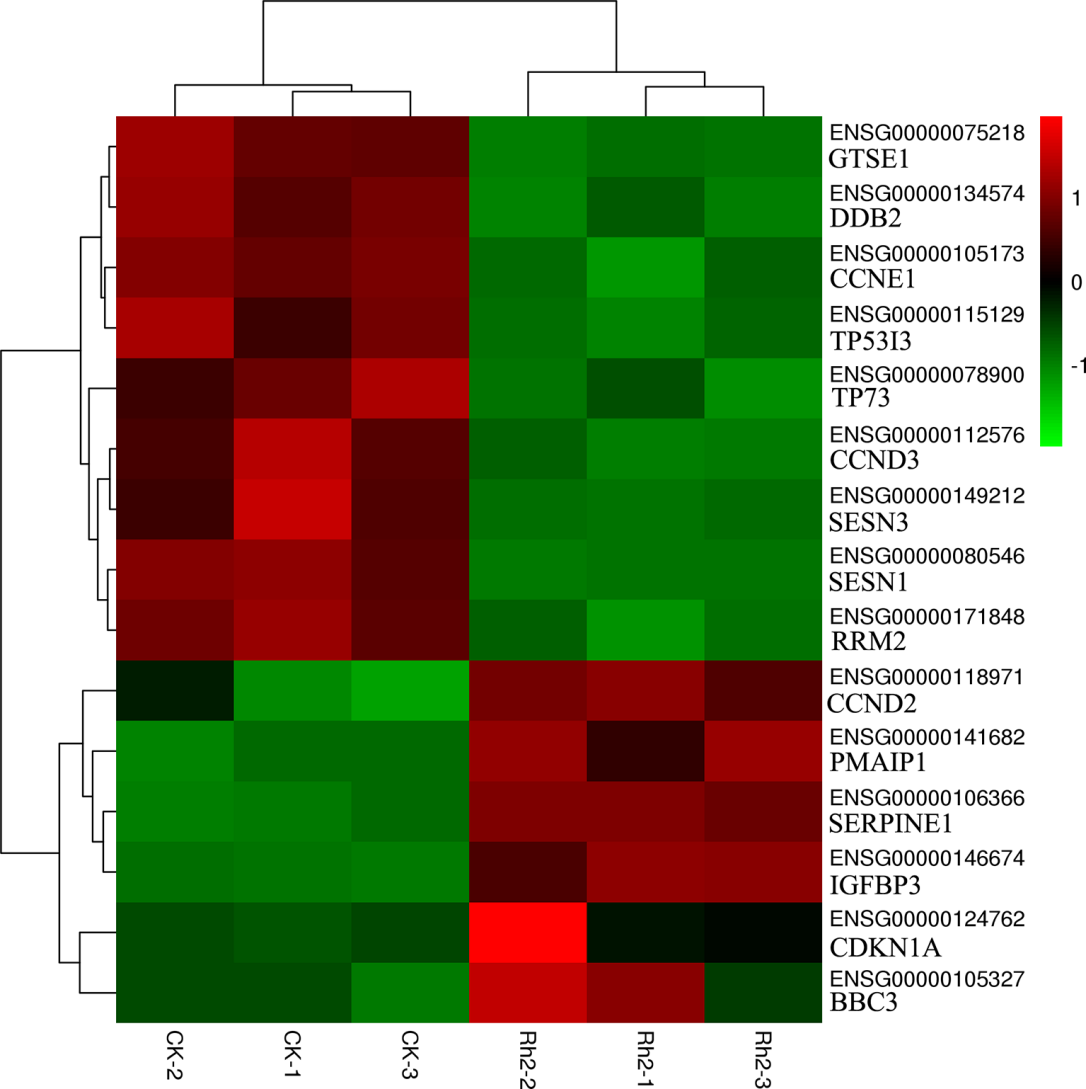
Heat map of representative DEGs in the apoptosis-related p53 signaling pathway by cluster analyses.

Based on the enrichment results of GO and KEGG, 15 DEGs belonging to the p53 signaling pathway were selected as representative DEGs for a heat map and are listed in **Table 4**. The color represents the relative expression of the genes. Red represents upregulation and green represents downregulation. As shown in **Figure 8** and **Table 4**, after Rh2 exposure, six genes (*PMAIP1*, *CDKN1A*, *CCND2*, *BBC3*, *SERPINE1*, and *IGFBP3*) were significantly upregulated, while nine (*SESN1*, *TP73*, *GTSE1*, *CCNE1*, *TP53I3*, *CCND3*, *DDB2*, *SESN3*, and *RRM2*) were significantly downregulated. In addition, the 10 DEGs annotated in the DNA replication pathway were all downregulated (**Tables 3** and **5**).

**Table 4:** p53 signaling pathway of DEGs in Rh2 exposed HepG2 cells.

Gene ID	Description	Gene symbol	Control fpkm	Rh2 fpkm	Regulated by Rh2
ENSG00000141682	Phorbol-12-myristate-13-acetate-induced protein 1	PMAIP1	1.97	4.73	Up
ENSG00000080546	Sestrin 1	SESN1	9.80	4.82	Down
ENSG00000078900	Tumor protein p73	TP73	1.90	0.73	Down
ENSG00000075218	G2 and S-phase expressed 1	GTSE1	14.68	6.41	Down
ENSG00000105173	Cyclin E1	CCNE1	11.61	5.62	Down
ENSG00000115129	Tumor protein p53 inducible protein 3	TP53I3	17.37	8.57	Down
ENSG00000124762	Cyclin dependent kinase inhibitor 1A	CDKN1A	34.89	72.18	Up
ENSG00000112576	Cyclin D3	CCND3	7.79	3.37	Down
ENSG00000118971	Cyclin D2	CCND2	0.23	0.65	Up
ENSG00000105327	BCL2 binding component 3	BBC3	13.20	34.10	Up
ENSG00000134574	Damage specific DNA binding protein 2	DDB2	46.41	20.76	Down
ENSG00000149212	Sestrin 3	SESN3	0.43	0.05	Down
ENSG00000171848	Ribonucleotide reductase regulatory subunit M2	RRM2	58.41	26.81	Down
ENSG00000106366	Serpin family E member 1	SERPINE1	328.87	732.31	Up
ENSG00000146674	Insulin like growth factor binding protein 3	IGFBP3	17.93	56.83	Up

**Table 5:** DNA replication pathway of DEGs in Rh2 exposed HepG2 cells.

Gene ID	Description	Gene symbol	Control fpkm	Rh2 fpkm	Regulated by Rh2
ENSG00000105486	DNA ligase 1	LIG1	24.78	11.14	Down
ENSG00000132646	Proliferating cell nuclear antigen	PCNA	160.84	45.83	Down
ENSG00000014138	DNA polymerase alpha 2, accessory subunit	POLA2	9.98	2.92	Down
ENSG00000073111	Minichromosome maintenance complex component 2	MCM2	106.54	37.80	Down
ENSG00000112118	Minichromosome maintenance complex component 3	MCM3	117.36	41.86	Down
ENSG00000104889	Ribonuclease H2 subunit A	RNASEH2A	22.45	9.86	Down
ENSG00000104738	Minichromosome maintenance complex component 4	MCM4	57.87	26.58	Down
ENSG00000100297	Minichromosome maintenance complex component 5	MCM5	71.27	24.30	Down
ENSG00000076003	Minichromosome maintenance complex component 6	MCM6	9.11	3.89	Down
ENSG00000106399	Replication protein A3	RPA3	15.07	4.64	Down

### RT-qPCR Verification of DEGs

**Figure 9:**
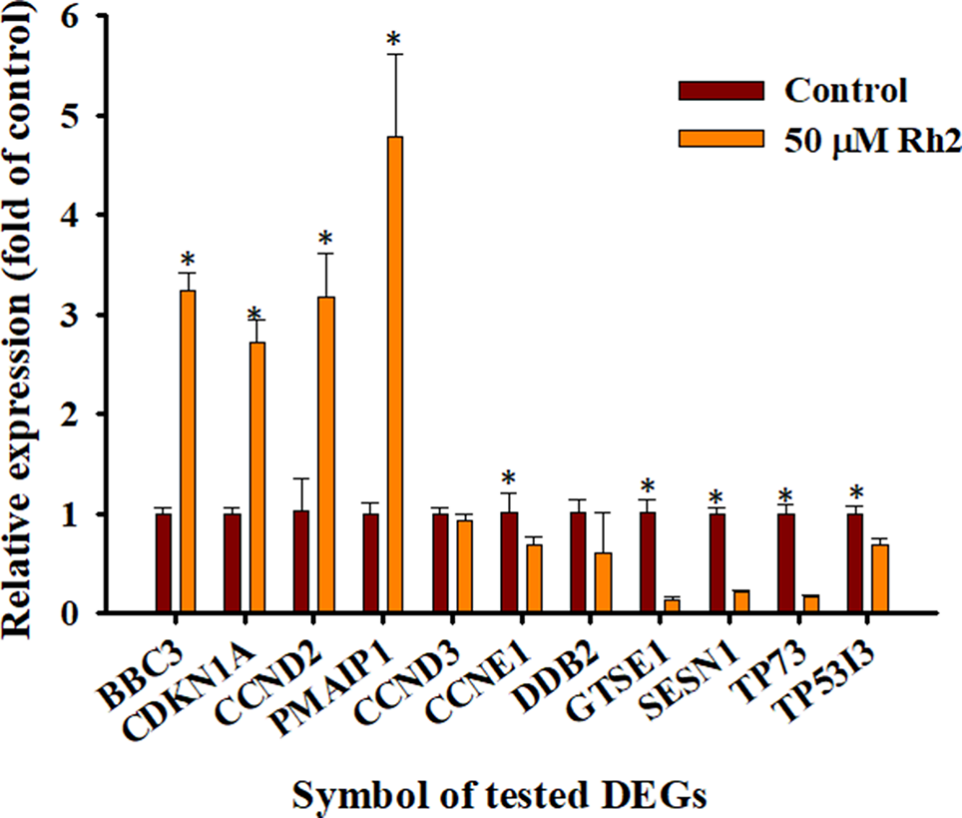
RT-qPCR verification of the selected DEGs. The mRNA expression levels of the test DEGs in the CK vs. Rh2 groups were quantified using RT-qPCR and the housekeeping gene GAPDH was used to normalize the relative expression level. Within the same gene, *p < 0.05 vs. control group.

RT-qPCR analyses were used to verify the accuracy of the transcriptome sequencing data. Eleven DEGs in the p53 signaling pathway were selected for qPCR analyses, including *PMAIP1*, *SESN1*, *TP73*, *GTSE1*, *CCNE1*, *TP53I3*, *CDKN1A*, *CCND3*, *CCND2*, *BBC3*, and *DDB2*. As shown in **Figure 9**, the DEGs *PMAIP1*, *CDKN1A*, *CCND2*, and *BBC3* were significantly upregulated, while *SESN1*, *TP73*, *GTSE1*, *CCNE1*, *TP53I3*, and *DDB2* were significantly downregulated on qPCR analyses. The expression of CCND3 was slightly downregulated. This differs slightly from the selection criteria (FC ≥ 2, FDR< 0.05) for DEGs. In general, the relative expression of the selected DEGs showed similar tendencies to the RNA-Seq results (**Tables 3** and **4**), indicating that the sequencing results were reliable.

## Conclusions

We demonstrated that Rh2 can inhibit proliferation and induce apoptosis in HepG2 cells. Investigations of the possible mechanism of the anti-proliferation effect revealed that Rh2 induced marked apoptosis that was related to changes in mRNAs in the p53 signaling pathway. Additional studies are currently underway to investigate its overall *in vivo* anticancer effect and potential mechanisms.

## Data Availability Statement

The data generated for this study can be found in the Sequence Read Archive, accession number PRJNA548682.

## Author Contributions

WH contributed to design of the study. JiZ, WL, QY, YC and JinZ performed all the experiments. JiZ and WH wrote the manuscript. JiaZ and GF contributed to manuscript revision. All authors read and approved the submitted version.

## Funding

This study was financially supported by National Natural Science Foundation of China (31600281), Huai'an 2017 Annual Promotion Project for Science and Technology International Cooperation (HAC201702), Natural Science Foundation of Jiangsu Province (BK20171269), 333 Project of Jiangsu Province (BRA2017241), and Natural Science Foundation of the Jiangsu Higher Education Institutions of China (18KJB320001).

## Conflict of Interest

The authors declare that the research was conducted in the absence of any commercial or financial relationships that could be construed as a potential conflict of interest.
